# Copper-2 Ingestion, Plus Increased Meat Eating Leading to Increased Copper Absorption, Are Major Factors Behind the Current Epidemic of Alzheimer’s Disease

**DOI:** 10.3390/nu7125513

**Published:** 2015-12-02

**Authors:** George J. Brewer

**Affiliations:** Human Genetics, University of Michigan, 3820 Gensley Road, Ann Arbor, MI 48103, USA; brewergj@umich.edu; Tel.: +1-734-761-7970

**Keywords:** Alzheimer’s disease, copper-2, copper plumbing, copper supplement pills, Alzheimer’s disease epidemiology

## Abstract

It has become clear that copper toxicity is playing a major role in Alzheimer’s disease; but why is the brain copper toxicity with cognition loss in Alzheimer’s disease so much different clinically than brain copper toxicity in Wilson’s disease, which results in a movement disorder? Furthermore, why is the inorganic copper of supplement pills and in drinking water so much more damaging to cognition than the organic copper in food? A recent paper, which shows that almost all food copper is copper-1, that is the copper-2 of foods reverts to the reduced copper-1 form at death or harvest, gives new insight into these questions. The body has an intestinal transport system for copper-1, Ctr1, which channels copper-1 through the liver and into safe channels. Ctr1 cannot absorb copper-2, and some copper-2 bypasses the liver, ends up in the blood quickly, and is toxic to cognition. Humans evolved to handle copper-1 safely, but not copper-2. Alzheimer’s is at least in part, a copper-2 toxicity disease, while Wilson’s is a general copper overload disease. In this review, we will show that the epidemiology of the Alzheimer’s epidemic occurring in developed, but not undeveloped countries, fits with the epidemiology of exposure to copper-2 ingestion leached from copper plumbing and from copper supplement pill ingestion. Increased meat eating in developed countries is also a factor, because it increases copper absorption, and thus over all copper exposure.

## 1. Introduction

For some time it has been clear that copper toxicity plays a major role in Alzheimer’s disease (AD). The studies published in multiple papers of a group in Italy led by Dr. Rosanna Squitti have shown how the “free copper” pool of the blood (the copper that can become toxic if the pool is expanded) is intimately tied to AD pathogenesis [1,2,3,4]. We will discuss this area in more detail later. However, a major question has been why the brain toxicity of copper in AD manifests itself in cognition loss, while the brain toxicity of copper in Wilson’s disease, another disease of copper toxicity, manifests itself as a movement disorder with no effect on cognition?

Second, it has been clear for some time that inorganic copper, the kind of copper found in drinking water and in supplements pills, is much more toxic and damaging to cognition than the organic copper found in foods. This will also be covered in detail later. However, in Wilson’s disease, all kinds of copper are toxic. Why the difference?

A recent paper, published by Ceko *et al.* in the December 2014, issue of Food Chemistry [5] gives sudden clarity, and answers these questions. These studies also give us sudden insight as to how to prevent most cases of AD. These authors studied the speciation of copper in a number of foods and in tap water. As expected, the copper in tap water was divalent copper, called here copper-2. However, a surprising and unexpected finding was that copper in food was primarily monovalent copper, or copper-1. The finding was surprising because it had always been assumed that food copper was a combination of copper-1 and copper-2. The reason for this assumption is that in living animal or plant tissue, both valence forms of copper exist, forming a redox doublet and allowing the catalysis of many redox reactions critical to life. Apparently, at death or harvest, in the absence of oxygen transport, the copper-2 is reduced to copper-1.

These findings give clarity and answers to both of the questions asked earlier. First the question of why the brain copper toxicity in AD affects cognition, but not movement control and brain toxicity in Wilson’s disease does the opposite. It is because AD brain copper toxicity is primarily caused by copper-2, while Wilson’s is a general copper overload disease with copper-2 playing no special role. It can be thought of as a copper-1 toxicity disease. Second, the question of why inorganic copper from drinking water and supplement pills is much more damaging to cognition than organic copper. It is because inorganic copper is all copper-2, while organic copper is all copper-1.

However, additional studies from the Squitti group [6,7,8,9] show that being a carrier of an ATP7B allele increases the risk for AD. ATP7B is the Wilson’s disease gene, and carriers have a slight increase in body copper load. Thus, this finding means that a lifetime of exposure to mildly increased copper load can increase risk of AD. Can this finding, too, be tied to the AD epidemic of the last century in developed countries? The answer is yes. Meat eating is greatly increased in developed countries *versus* undeveloped countries because of the expense of meat. Meat eating has also increased in developed countries over the last century because of the expansion of beef cattle, hogs, and other meat sources. Furthermore, the key to understanding the significance of this is to know that copper is much better absorbed from meat than it is from vegetable foods. Thus, the increase ingestion of meat in developed countries in the last century, increases overall copper absorption, and makes carriers of ATP7B alleles at an increased risk.

These revelations open the door to how to prevent much of AD, simply by avoiding ingestion of copper-2 in drinking water and in supplement pills, and reducing meat eating. In the rest of this review to follow, what has been introduced here will be embellished, and considerably more information will be added to the story of copper toxicity in AD.

## 2. The Epidemiology of Alzheimer’s Disease over the Last Two Centuries

The epidemiology of AD over the last two centuries make it very clear that there is a new environmental factor or factors arising over the last century in developed countries that is, or are, causing an explosion in the prevalence of AD, that is, an AD epidemic. It is very important to understand this, because if it is true and this new environmental factor or factors can be identified, it may be possible to eliminate it or them, and greatly reduce the prevalence of AD. This would make a critical difference to tens of millions of people around the world. 

So what are the epidemiologic facts that lead to the conclusion that there is a new environmental factor of factors in developed countries?

First, there is universal agreement that currently we have a very high prevalence of AD in developed countries. For example, in the US, 10% of those 60 years and older, 20% of those 70 and over, and 30% of those 80 and over have AD.

Second, there is good documentation that undeveloped countries do not share in these high prevalence figures [10]. For example in rural India, in those aged 65 and over, AD prevalence is 1.07% [11], and in Nigeria, Africa, in those aged 65 to 74, AD prevalence is 0.52% [12] and in those aged 75–84 was 1.69%. Interestingly in the Nigerian study, the prevalence of AD in African-Americans in Indianapolis US, in those aged 75–84 was 8.02%. This five-fold higher prevalence of AD in those with the same African ethnic background shows the strong effect of living in a developed country on increasing AD prevalence.

Third, the data strongly indicate that the prevalence of AD in the 1800s and early 1900s in countries that are now “developed” was quite low, probably at about the 1% level currently seen in undeveloped countries. This is somewhat controversial, but the data are relatively good. They are in part the observations of prestigious clinicians practicing during these periods that an AD-like dementia was not seen [13,14,15], and observations of pathologists that amyloid plaques and neurofibrillary tangles, hallmarks of AD brain pathology, were not seen in autopsied brains during these periods [16]. This area has been reviewed in more detail elsewhere [17]. One argument that the reason for the relative paucity of AD in these earlier periods is, that since AD is a disease of aging, there were not enough elderly people back then to make the occurrence of AD apparent. However, this argument is invalid because in 1911, half the population of France was 60 or older [18], and in 1900 in the US, 3,600,000 people were age 60 or older, enough to generate 360,000 cases at today’s rate. A second argument is that AD was simply viewed as normal aging, and the people with AD were not thought of as having a specific disease. However, while this conceivably could have been a factor in the clinicians not being aware of AD, it would not explain why the pathologists did not see plaques and tangles in brain at autopsy. This controversy could be settled if there is brain tissue still available from 1850 to 1925, in those aged 60 and over, and examined to see if modern pathologic techniques reveal a significant prevalence of plaques and tangles. It is important to settle this controversy if possible, because if it becomes clear that the prevalence of AD is now much higher in developed countries than in times past, it makes it certain that the environment has changed in developed countries, and increases the urgency to identify the environmental change, to see if it can be rectified.

Here it will be assumed that the data are convincing that the current prevalence in developed countries is much higher now than in the past, and that therefore, the search for causative environmental change is urgent.

## 3. Risk Factors for AD

It is proposed here that copper-2 ingestion is a major new environmental risk factor for AD. The data supporting this and the details of this proposal will be given in a later section, but first it is relevant to review the other known risk factors for AD.

Age is a major risk factor, but of course it is not absolute, because the majority of elderly people do not develop AD. There are certain genes that increase the risk. Having the apolipoprotein E4 allele increases risk [19] while the E3 allele is neutral, and the E2 allele seems to offer protection against AD. Having certain alleles of the hemochromatosis [20], transferrin [21], and ATP7B [6,7,8,9] genes seems to increase risk. The basis for concluding that certain alleles of these genes increase the risk of AD is the increased prevalence of these alleles in AD patients compared to the non-AD population. Having an increase homocysteine blood level also increases risk of AD [22]. Furthermore, Grant [23] has shown that AD prevalence in countries around the world correlates with dietary fat intake, with the implication that increased fat intake is a risk factor for AD. Meat eating, because of economic factors, as mentioned before, has greatly increased in developed countries, with a consequent great increase in fat intake. Furthermore, as mentioned above it is now proposed that ingestion of copper-2 is a major risk factor.

It is relevant to review the known risk factors, because the question needs to be asked, would it be expected for any of them to greatly increase in the last century in developed countries? Average age has increased, but as mentioned in the Introduction, there were elderly people in 1900–1911, but very little AD. It would not be expected that there would be much change in the allele frequency of the genes that affect risk. Homocysteine levels might be higher as a result of dietary changes, but the change would not be expected to be large. However, dietary fat intake has probably increased considerably in developed countries, as a result of increased meat eating. The increased meat eating also increases general copper exposure, because of better absorption of copper from meat compared to vegetable foods, as mentioned earlier. Furthermore, finally, as we will discuss in another section, copper-2 intake has greatly increased as a result of copper plumbing and taking supplement pills containing copper.

Thus, in summary of risk factors, increased meat intake and copper-2 can be identified as greatly increased in developed countries, but not undeveloped countries, from 1900 until now.

## 4. The Evidence that Alzheimer’s Disease Is in Part a Copper Toxicity Disease

In this section the focus will not be on copper-2 toxicity in AD, but rather on the proposition that AD pathogenesis is intimately tied to copper toxicity in general, without regards to copper valence. As stated in the Introduction, Dr. Rosanna Squitti and her group in Italy have led the way in showing that AD pathogenesis is closely tied to the size of the free copper pool in the blood of AD patients. Before getting into the evidence, the blood free copper should be defined so what is meant by this term is clearly understood. The copper in the blood can be thought of as in two pools. One pool is the copper covalently bound to ceruloplasmin (Cp). This pool includes the majority of the blood copper, 80% to 90% depending on how Cp is measure. The rest of the copper in the blood is loosely bound to proteins such as albumin and other molecules, and is freely available for use by blood tissues. This pool is called non-Cp copper, or blood “free copper”, although it is not really free, just loosely bound. Here this pool will be called the blood free copper.

Blood free copper, if the pool is increased in size, becomes toxic copper. The best example of that is Wilson’s disease, an inherited disease of copper accumulation and copper toxicity [24]. Before the disease is treated, the blood free copper is greatly increased, and there is great copper toxicity. During treatment to lower body copper, the blood free copper gradually shrinks back to normal, and copper toxicity is abated.

What the Squitti group has shown in AD is that: (1) The blood free copper pool in AD patients is significantly increased in size compared to age-matched controls [1]; (2) The size of the blood free copper pool in AD patients correlates with measures of cognition, that is, the higher the blood free copper pool, the poorer the cognition [2]; (3) The blood free copper pool predicts the rate of cognition loss over time, that is, the higher the blood free copper cool the greater the rate of cognition loss [3]; (4) The size of the blood free copper pool predicts the risk for the conversion of mild cognitively impaired (MCI) patients, the precursor state to AD, to full AD, that is, the greater the free copper pool, the higher the risk of conversion [4].

These studies show first, that the toxic pool of copper, the blood free copper, is increased in AD [1]. Then second, they show that the extent of the increase in this toxic cool free copper is closely tied to the damage in AD, loss of cognition [2,3,4]. These studies confirm that AD can be identified, at least in part, as a copper toxicity disease.

The studies of the Squitti group are strong evidence of copper toxicity, but there is more evidence. James *et al.* [25] have studied AD patients at autopsy, and compared to controls find increased “labile” (or free) copper in AD brains, and increase oxidative damage from copper. Additionally, there are studies from China by Shen *et al.* [26]. They find that the soil concentration of copper correlates positively with the prevalence of AD across the provinces of Mainland China. For example, the prevalence of AD is 2.6 times as high if the soil concentration of copper is 60–80 parts per million (ppm), as it is if soil concentration is 20–40 ppm. 

Finally, there are various pieces of evidence related to copper-2 specific toxicity, to be covered in the next section. However, this section summarizes more than adequate data to show that AD is, at least in part a copper toxicity disease.

## 5. Special Cognitive and Alzheimer’s Disease-Related Toxic Effects of Inorganic Copper (Copper-2)

The first clue that inorganic copper might have very specific and sensitive toxicity in AD came from a 2003 paper by Sparks and Schreurs [27]. There is an interesting background to their findings which shows scientific detective work at its best, and resulting in a very important finding. Larry Sparks had developed an excellent AD animal model in rabbits, using cholesterol feeding, in his W. Virginia laboratory. The rabbits developed the amyloid plaques in the brain typical of AD, and memory loss, as occurs in AD. When he moved his laboratory to Arizona, cholesterol feeding to rabbits of the same breed he used in W. Virginia no longer produced the AD-like disease. After considerable effort at trying to find what was different, Dr. Sparks finally realized that in W. Virginia they used tap water, while in Arizona distilled water was being used. After experimenting with various substances found in the tap water, they discovered the key substance was trace amounts of copper. They reported in their paper that adding 0.12 ppm of copper to distilled drinking water greatly enhanced amyloid plaques in the brain and memory loss in the rabbits [27]. This is a tiny amount of copper, and this amount added to food would have no toxic effects. For example, animal chow runs 3–6 ppm concentration of copper. One could change the animal chow from 3 to 6 ppm, a 25 times greater increase in copper than the 0.12 ppm added to the drinking water, and it would have no toxic effects. The effect of 0.12 ppm copper on enhancing AD-type toxicity was shown in other AD animal models, including the mouse model, by the Sparks group [28]. These findings were also confirmed by another group, in a mouse model [29]. For reference, the amount of copper allowed in human drinking water in the US by the Environmental Protection Agency (EPA) is 1.3 ppm, ten times the amount found toxic in the animal model studies.

One would have thought that these extraordinary findings, especially after their confirmation in several different animal models, and in two independent laboratories, would have attracted the attention of the AD scientific community, especially since 80% plus of homes in developed countries have copper plumbing, and there has been no data to indicate how much copper is leached from copper plumbing into drinking water. However, mysteriously this has not been the case. Nor have these findings attracted the attention of regulatory agencies such as the EPA in the US, which allow potentially toxic amounts of copper in human drinking water.

The second piece of evidence that inorganic copper had very specific toxicity on cognition came from a 2006 paper by Morris and colleagues [30]. They studied a large Chicago population for intake of various nutrients, and change in cognition over time. They found that those in the highest quintile of copper intake, who were there because they ingested a supplement pill containing copper, if they also ate a high fat diet, lost cognition at six times the rate of other groups. This is another extraordinary finding. It indicates that in those taking a supplement pill containing copper, as many people in developed counties do, if they also eat a high fat diet, again as many people in developed countries do, cognition is lost at six times the normal rate. One would think that regulatory agencies, such as the FDA in the US, which has regulatory authority over toxicity in supplements, would show interest in these results, at least to the point of requiring more studies. However, mysteriously, no interest has been shown.

Of course, the copper in supplement pills, like that in drinking water, is copper-2. So what is it about inorganic copper, copper-2, that makes it uniquely toxic to cognition, and potentially causative of AD? One clue was given in studies many years ago when radiolabeled copper (copper-64), as an inorganic salt, was given to Wilson’s disease patients to evaluate whether zinc therapy was effectively blocking copper absorption. In baseline studies, before zinc therapy, when copper-64 was given orally, again as an inorganic salt, 15%–25% of the label showed up immediately in the blood, in 1 to 2 h [31]. In contrast, if food copper was labeled with a longer-lived isotope of copper (copper 64 has a 12 h half-life), the radiolabeled copper would not appear in the blood for one or two days, and would appear covalently bound to a protein, such as Cp, secreted into the blood by the liver. What these studies show is that food (organic) copper first passes through the liver, and is put into safe channels, while some of the inorganic copper bypasses the liver and shows up immediately in the blood, expanding the blood free copper pool.

Why is inorganic copper absorbed differently than the organic copper of food? The recent studies of Ceko *et al.* [5], discussed in the Introduction, now explain everything. As discussed in the Introduction, they found that food copper is primarily copper-1, this finding coming as a surprise, as also discussed in the Introduction. What this means is that mammals, including humans, evolved ingesting copper-1, and not copper-2. As a consequence, there is an intestinal transport system, called Ctr1, specific for transporting copper-1 [32]. Copper transported through this system goes to the copper chaperone ATOX1, which gives the copper off to the enzyme ATP7A, which loads it into secretory vesicles that are transported through the blood to the liver. Copper-2 cannot be transported through Ctr1 unless reduced to copper-1. In the absence of reductants, it can be transported through the divalent cation transporter, or by diffusion. Some of this copper-2 bypasses the liver, and is absorbed directly into the blood, where it is quite toxic to cognition.

Summarizing what has been shown in this section, first it was shown that tiny amounts of inorganic copper in drinking water greatly enhanced AD pathology and memory loss in AD animal models [27,28,29]. Second it was shown that ingestion of inorganic copper in supplement pill form was associated with large losses of cognition in humans [30]. Third, it was shown that a portion of inorganic copper is absorbed differently than food (organic) copper, bypassing the liver and appearing immediately in the blood [31]. Lastly, the explanation for this difference in absorption was shown. Food copper is all copper-1 [5], and there is a specific intestinal transport system for it [32], causing it to be metabolized through the liver, while humans did not evolve to handle copper-2 safely, and some of it bypasses the liver, and is toxic to cognition.

## 6. Sources of Copper-2 Ingestion

There are two major sources of copper-2 ingestion that have been identified. One of these is ingestion of supplement pills containing copper-2. Most multivitamin/multimineral supplement pills contain copper-2, and it is estimated that at least a third of the US population takes one of these pills. Probably, a similar number takes them in other developed nations.

The second source of copper-2 ingestion is from drinking water in homes with copper plumbing, where copper is leached from the plumbing into the drinking water. It is a fair question to ask, is it known how much copper is actually leached from copper plumbing into drinking water? The answer is yes.

In studies of household drinking water to evaluate the copper content of drinking water of Wilson’s disease patients, 280 samples of drinking water from all over N. America was studied [33]. It was found that about one third had copper content 0.1 ppm or higher, the level toxic in AD animal models, about one third were below 0.01 ppm, a level deemed safe, and about one third were between these levels, and of unknown safety. Thus, one- to two-thirds of samples of household drinking water had copper levels of known or possible toxicity, if the animal models are a guide.

A third possible source of copper-2 ingestion is copper sulfate residues left on fruits, some nuts and vegetables after use as a pesticide. It is unknown if this copper is completely or almost completely removed by washing the produce.

## 7. How Copper-2 Ingestion Fits into Being the Major Environmental Culprit Triggering Alzheimer’s Disease, and Thereby the Major Cause of the Alzheimer’s Disease Epidemic

Since in the previous section it was documented that copper leaches into drinking water from many homes with copper plumbing in high enough concentrations to be toxic to cognition, it is informative to compare the epidemiology of AD prevalence with the epidemiology of copper plumbing use in developed countries since 1900. With respect to AD, the disease was quite rare in the 1800s and early 1900s, as we discussed in the section on AD epidemiology. It gradually increased in frequency until about 1950, and then rapidly increased in frequency and exploded in the last 50 years. Copper plumbing began to be used in the early 1900s, and then was curtailed by two World Wars, and then exploded in use after 1950, such that now, as an example, 80%–90% of US homes have copper plumbing.

Thus, the epidemiology of AD and copper plumbing use fit very well with the hypothesis that copper in drinking water, leached from copper plumbing used in developed countries, is a major triggering factor for AD. There is an interesting fact that adds to the credibility of this hypothesis. Japan is a developed country but has a low prevalence of AD [34]. Furthermore, Japan has shunned the use of copper plumbing, possibly for fear of toxicity. However, when Japanese migrate to Hawaii, where copper plumbing is used, their prevalence of AD increases to that of other developed countries [35].

The second source of copper-2 ingestion, that of ingestion of supplement pills containing copper, also fits with the epidemiology of the AD epidemic. Supplement pills were rarely used by people before 1945, but after World War 2, affluence and health consciousness increased, leading to a large increase in the use of supplement pills, concurring with the large increase in AD prevalence.

In discussing risk factors for AD in a previous section, it was stated that the only two known risk factors that would have undergone major increase in the period that AD prevalence was increasing rapidly were dietary meat intake and copper-2 intake. Here, it is believed that of these two, copper-2 ingestion is more important. That belief is based on the Japanese data cited in this section. When Japanese migrated to Hawaii, the prevalence of AD increased dramatically, concurrently with the switch to copper plumbing. While the dietary meat intake might have also gone up, there is no reason to believe it would have done so dramatically.

## 8. How Copper-2 Ingestion as a Trigger for Alzheimer’s Disease Fits into Current Theories of Alzheimer’s Disease Pathogenesis

The most popular theory of AD pathogenesis is called the amyloid cascade hypothesis [36]. A piece of protein called the amyloid precursor protein is clipped off by an enzyme called beta secretase. The piece is called beta amyloid. This process goes on the normal brain, but the function of the process is unclear. In the AD brain beta amyloid accumulates, possibly because it is formed faster, possibly because it is cleared more slowly, or possibly some agent, such as copper, increases aggregation faster than clearance. In the AD brain the accumulating beta amyloid aggregates, forming the amyloid plaques so characteristic of the pathology of the AD brain. According to this theory, these plaques are damaging to neurons, possibly because they emit damaging oxidant radicals, particularly if they bind metals such as iron or copper. This theory of AD pathogenesis is supported by the consistent presence of amyloid plaques in the AD brain. A key role for amyloid involvement in AD causation is also provided by mutations in the amyloid precursor protein leading to early onset AD [37].

The copper-2 hypothesis fits into the amyloid cascade hypothesis in two ways. First, copper is known to cause or enhance aggregation of beta amyloid into plaques [38]. Second, copper is known to bind to plaques and cause release of damaging oxidant radicals [39]. To be clear, under the amyloid cascade hypothesis it is not proposed that copper-2 is causal of AD, but rather that it is a major triggering agent causing beta amyloid to aggregate and be toxic.

Not all agree with the amyloid cascade hypothesis. A second theory, which might be called the oxidant damage theory, for AD pathogenesis has developed. Under this theory, the primary event is oxidant damage [40]. This theory holds that beta amyloid is protective, and accumulates to protect the neurons from oxidant damage. As it accumulates, it aggregates into plaques. Under this theory, the plaques are the result of damage, the proverbial cart, while the oxidant damage is the cause, the proverbial horse [41]. Under this theory, copper-2 could be a causal agent, causing the original oxidant damage. Beta amyloid is known to reduce copper-2 to copper-1, so its protective role might be this reductant step.

In summary, whichever pathogenic theory for AD turns out to be correct, copper-2 could play a key role, as a triggering agent for beta amyloid aggregation in the amyloid cascade hypothesis, or as a causal agent in the oxidant damage hypothesis.

## 9. Is General Copper Load, Irrespective of Copper Valence, a Risk Factor for Alzheimer’s Disease?

So far in this review, we have emphasized the role of divalent copper, or copper-2, in triggering AD, and being a major cause of the current major epidemic of AD. However, there is also evidence that general copper overload could be a risk factor. The best evidence for that is a series of papers by the Squitti group, in which they find that carriers of a variant ATP7B allele are at increased prevalence in AD patients compared to controls [6,7,8,9]. ATP7B is the Wilson’s disease gene, and patients with both alleles mutated have accumulation of large amounts of copper, and copper toxicity. It is a general copper overload disease, but there are no specific effects on cognition. Patients with the neurologic movement disorder of Wilson’s disease often have behavioral disturbance, some of them severe. The strange behavior of these patients can give the impression of dementia, but it is not true dementia. With therapy, and improvement in the neurological picture the behavioral disturbance also improves, and the appearance of dementia disappears. Patients who are heterozygous carriers of one mutant allele of ATP7B have only mild copper accumulation that does not require treatment. Thus, if homozygous ATP7B causes large copper overload, but no specific effect on cognition, why should the Squitti group find a higher prevalence of ATP7B heterozygous variants in the AD population [6,7,8,9], suggesting that such carriers have a higher risk for AD in spite of only mild increase in copper burden?

A reasonable explanation is that most Wilson’s disease patients either die at a relatively young age, or are treated to remove the excess copper at a relatively young age. Thus, they are not exposed to the high copper burden during aging, and aging, of course, is a very important risk factor. Heterozygous carriers of a variant ATP7B allele are never treated and are exposed to a lifetime of somewhat elevated copper, including free copper, levels. This appears to be a significant risk factor as it is coupled with aging, in aging patients.

If mild general copper overload is a risk factor, as the data suggest [6,7,8,9], then it raises the question of whether dietary changes in developed countries could be a factor in the AD epidemic? The one that seems most likely is increased meat eating, which is increased in developed countries because increased affluence leads to increased meat eating. The effect of increased meat eating producing an increased fat intake, a likely AD risk factor, has already been mentioned. However, increased meat eating would also have a significant effect on increasing copper absorption, because copper is much better absorbed from meat than vegetable foods [42].

All of this suggests that the AD epidemic may be a coming together of copper-2 exposure, increased dietary fat from increased meat eating, increased copper absorption from the increased dietary meat, and in some patients increased copper levels from carrying an ATP7B allele. If this analysis is correct, it indicates that both exposure to copper-2 and increased general copper overload contribute to AD risk. The latter suggests that dietary change, particularly a reduction in meat eating, could be helpful.

## 10. Recommendations for Avoiding Copper-2 Ingestion and a High Fat, High Meat, Diet to Abort the Epidemic of Alzheimer’s Disease

There are two key steps to avoid copper-2 ingestion. The first is avoiding supplement pills containing copper, and the second is to avoid regular ingestion of drinking water with toxic levels of copper.

To avoid ingestion of supplement pills containing copper, all that is necessary is to look on the label of supplement pills about to be purchased, or which already have been purchased and are in the home. If the label states that copper is present, in any amount, do not make the purchase, and if already purchased, throw the bottle away. Almost all multivitamin/multimineral and multimineral preparations currently for sale contain copper. Multivitamin preparations without minerals are available. If a mineral is needed, such as iron or calcium, it can be purchased as an individual item.

The rationale for including copper in multimineral supplements is that some scientists have argued that many people in the general population are copper deficient. There are some patients, such as those with extensive intestinal surgery or malabsorption syndromes, that require supplemental copper, but these patients are well identified, and should be supplemented. Unfortunately, the only copper supplement pills available are the toxic copper-2. However, unidentified copper deficiency in the general population is exceedingly rare. People in the general population do not require copper supplementation, but more than that, their cognition is at great risk of being harmed if they take copper-2 supplements.

Regarding copper in drinking water, everyone should test their water for copper levels. They should do this even if they do not have copper plumbing, because sometimes source water is high in copper. Many companies offer this testing service. When collecting the sample, the water should be allowed to run for five minutes. So-called “first draw” water may have higher copper as a result of sitting in the plumbing for several hours. If the copper level is 0.01 ppm or below, it is safe. If it is higher than that, the copper plumbing need not be torn out. A device can be attached to the tap used for drinking and cooking, such as a reverse osmosis device, which will remove the copper to a safe level.

There is a third possible source of copper-2 ingestion of unknown importance. This is ingestion of copper-2 from copper sulfate left on some fruits and other produce when it is used as a pesticide. Probably most people wash these products before consumption, but it is not known how effective this is in removing all of the copper.

In addition to copper-2, it appears likely that a high fat, high meat, diet is now a contributor to triggering AD [24]. Therefore, it would probably be a good idea to reduce dietary fat and meat intake. Since most fat in the diet is consumed as meat, the simplest way to reduce dietary fat is to reduce meat consumption. Reducing meat intake would also reduce copper absorption, another possible risk factor as discussed in Section 9. Reducing meat intake is a healthy move anyways, because lowering meat consumption in one large study reduced all-cause mortality by 42% [43].

What would be the impact of eliminating copper-2 ingestion and reducing dietary meat intake? The best scenario would be to eventually reduce the AD prevalence in developed countries to the 1% to 1.5% prevalence seen currently in undeveloped countries in their elderly population. In other words, it is to be expected that a low prevalence of AD will exist because the other risk factors will occasionally come together to produce AD. However, the result of decreasing the current 20% of those 70% and over getting AD to 1%, in other words essentially eliminating the epidemic of the last century, would save tens of millions of people every year from getting this serious disease, one that slowly robs them of their humanity.

## 11. Recommendations for Government and Industry to Help Abort the Epidemic of Alzheimer’s Disease

The most important government action is for regulatory agencies, such as the FDA in the US, to examine the toxicity of copper-2 in various supplement pills. Here, the evidence seems relatively strong that this copper is toxic. That evidence is, first, the studies of Morris *et al.* [30] that show that ingestion of copper supplement pills is associated with a six-fold increase in cognition loss. Second, there is a study from the Iowa Women’s Health Study published by Mursu *et al.* [44], which shows that ingestion of copper supplement pills is associated with a 42% increase in overall mortality. Finally, there are the animal model studies [27,28,29], which show that tiny amounts of copper-2 in drinking water dramatically enhanced AD-like toxicity. If these data are deemed inadequate to take action, it is urged that these agencies should further investigate as necessary the toxic effects of copper-2, and take action to protect consumers as appropriate. If they find copper-2 toxic, then it should only be available by a doctor’s prescription (see recommendation below to industry to manufacture all copper in copper containing supplement pills to be copper-1).

The second most important government action is for regulatory agencies, such as the EPA in the US, to consider lowering allowable copper levels in drinking water to 0.01 ppm. This will help eliminate high copper-2 levels in the source water in some parts of the country. It will not help eliminate high copper-2 levels resulting from leaching of copper-2 from copper plumbing, but it will be at least help inform the consumer about what are safe levels of copper in drinking water.

It is recommended that industry consider ceasing to put potentially toxic copper-2 in supplement pills, whether regulatory agencies require it or not. They need to read the literature and realize they may be poisoning their customers. Second, it is recommended that industry manufacture a copper-1 containing pill, so that patients who require copper supplementation can take copper-1 rather than the toxic copper-2.

## 12. Summary and Conclusions

In summary, here it is proposed:

(1) That the current high prevalence of AD in developed countries is new, occurring within the last century, that the prior prevalence was around 1% in the elderly, similar to the current AD prevalence in undeveloped countries.
(2) That this means that some factor, or factors, is new in the environment of developed countries, causing the new epidemic of AD in developed countries.
(3) That the main new environmental factor is copper-2 ingestion, from drinking water and copper containing supplement pills, with a high fat, high meat, diet being a secondary factor.
(4) That if copper-2 ingestion ceases, and the fat and meat in the diet reduced, there will be a great decrease in AD prevalence, aborting the current epidemic.

In conclusion, if the above proposals, summarized under the rubric “the copper-2, high meat hypothesis”, are correct, and many people follow the recommendations, hundreds of millions of people could eventually be saved from suffering this terrible disease. It appears the information on which the proposals are based is sound, so the copper-2, high meat hypothesis has an excellent chance of being correct. Thus, it may be prudent to act accordingly. As an example that may be relevant, those who stopped smoking when it was only a hypothesis that cigarette smoking caused lung cancer and other diseases, had already benefitted greatly by the time the hypothesis was found to be correct.

## References

[1] Squitti R., Pasqualetti P., dal Forno G., Moffa F., Cassetta E., Lupoi D., Vernieri F., Rossi L., Baldassini M., Rossini P.M. (2005). Excess of serum copper not related to ceruloplasmin in Alzheimer disease. Neurology.

[2] Squitti R., Barbati G., Rossi L., Ventriglia M., dal Forno G., Cesaretti S., Moffa F., Caridi I., Cassetta E., Pasqualetti P. (2006). Excess of nonceruloplasmin serum copper in AD correlates with MMSE, CSF β-amyloid, and h-tau. Neurology.

[3] Squitti R., Bressi F., Pasqualetti P., Bonomini C., Ghidoni R., Binetti G., Cassetta E., Moffa F., Ventriglia M., Vernieri F. (2009). Longitudinal prognostic value of serum “free” copper in patients with Alzheimer disease. Neurology.

[4] Squitti R., Ghidoni R., Siotto M., Ventriglia M., Benussi L., Paterlini A., Magri M., Binetti G., Cassetta E., Caprara D. (2014). Value of serum nonceruloplasmin copper for prediction of mild cognitive impairment conversion to Alzheimer disease. Ann. Neurol..

[5] Ceko M.J., Aitken J.B., Harris H.H. (2014). Speciation of copper in a range of food types by X-ray absorption spectroscopy. Food Chem..

[6] Bucossi S., Polimanti R., Mariani S., Ventriglia M., Bonvicini C., Migliore S., Manfellotto D., Salustri C., Vernieri F., Rossini P.M. (2012). Association of K832R and R952K SNPs of Wilson’s disease gene with Alzheimer’s disease. J. Alzheimers Dis..

[7] Squitti R., Polimanti R., Bucossi S., Ventriglia M., Mariani S., Manfellotto D., Vernieri F., Cassetta E., Ursini F., Rossini P.M. (2013). Linkage disequilibrium and haplotype analysis of the ATP7B gene in Alzheimer’s disease. Rejuvenation Res..

[8] Squitti R., Polimanti R., Siotto M., Bucossi S., Ventriglia M., Mariani S., Vernieri F., Scrascia F., Trotta L., Rossini P.M. (2013). ATP7B variants as modulators of copper dyshomeostasis in Alzheimer’s disease. Neuromol. Med..

[9] Bucossi S., Polimanti R., Ventriglia M., Mariani S., Siotto M., Ursini F., Trotta L., Scrascia F., Callea A., Vernieri F. (2013). Intronic rs2147363 variant in ATP7B transcription factor-binding site associated with Alzheimer’s disease. J. Alzheimers Dis..

[10] Ferri C.P., Prince M., Brayne C., Brodaty H., Fratiglioni L., Ganguli M., Hall K., Hasegawa K., Hendrie H., Huang Y. (2005). Global prevalence of dementia: A Delphi consensus study. Lancet.

[11] Chandra V., Ganguli M., Pandav R., Johnston J., Belle S., DeKosky S.T. (1998). Prevalence of Alzheimer’s disease and other dementias in rural India: The Indo-US study. Neurology.

[12] Hendrie H.C., Osuntokun B.O., Hall K.S., Ogunniyi A.O., Hui S.L., Unverzagt F.W., Gureje O., Rodenberg C.A., Baiyewu O., Musick B.S. (1995). Prevalence of Alzheimer’s disease and dementia in two communities: Nigerian Africans and African Americans. Am. J. Psychiatry.

[13] Strachey J., Freud A., Strachey A., Tyson A. (1966). 24 Volumes Entitled, The Standard Edition of the Complete Psychological Works of Sigmund Freud, Written between 1895 and 1939.

[14] Gowers W.R. (1888). A Manual of Diseases of the Nervous System.

[15] Osler W. (1910). Modern Medicine in Theory and Practice.

[16] Boyd W. (1938). A Textbook of Pathology: An Introduction to Medicine.

[17] Brewer G.J. (2012). Copper toxicity in Alzheimer’s disease: Cognitive loss from ingestion of inorganic copper. J. Trace Elem. Med. Biol..

[18] Waldman M., Lamb M. (2005). Dying for a Hamburger: Modern Meat Processing and the Epidemic of Alzheimer’s Disease.

[19] Miyata M., Smith J.D. (1996). Apolipoprotein E allele-specific antioxidant activity and effects on cytotoxicity by oxidative insults and β-amyloid peptides. Nat. Genet..

[20] Moalem S., Percy M.E., Andrews D.F., Kruck T.P., Wong S., Dalton A.J., Mehta P., Fedor B., Warren A.C. (2000). Are hereditary hemochromatosis mutations involved in Alzheimer disease?. Am. J. Med. Genet..

[21] Zambenedetti P., de Bellis G., Biunno I., Musicco M., Zatta P. (2003). Transferrin C2 variant does confer a risk for Alzheimer’s disease in caucasians. J. Alzheimers Dis..

[22] Seshadri S., Beiser A., Selhub J., Jacques P.F., Rosenberg I.H., D’Agostino R.B., Wilson P.W., Wolf P.A. (2002). Plasma homocysteine as a risk factor for dementia and Alzheimer’s disease. NEJM.

[23] Grant W.B. (1997). Dietary links to Alzheimer’s disease. Alzheimers Dis. Rev..

[24] Brewer G.J., Kasper D., Fauci A., Hauser S., Longo D., Jameson J.L., Loscalzo J. (2015). Wilson’s Disease. Harrison’s Principles of Internal Medicine.

[25] James S.A., Volitakis I., Adlard P.A., Duce J.A., Masters C.L., Cherny R.A., Bush A.I. (2012). Elevated labile Cu is associated with oxidative pathology in Alzheimer disease. Free Radic. Biol. Med..

[26] Shen X.L., Yu J.H., Zhang D.F., Xie J.X., Jiang H. (2014). Positive relationship between mortality from Alzheimer’s disease and soil metal concentration in mainland China. J. Alzheimers Dis..

[27] Sparks D.L., Schreurs B.G. (2003). Trace amounts of copper in water induce β-amyloid plaques and learning deficits in a rabbit model of Alzheimer’s disease. Proc. Natl. Acad. Sci. USA.

[28] Sparks D.L., Friedland R., Petanceska S., Schreurs B.G., Shi J., Perry G., Smith M.A., Sharma A., Derosa S., Ziolkowski C. (2006). Trace copper levels in the drinking water, but not zinc or aluminum, influence CNS Alzheimer-like pathology. J. Nutr. Health Aging.

[29] Singh I., Sagare A.P., Coma M., Perlmutter D., Gelein R., Bell R.D., Deane R.J., Zhong E., Parisi M., Ciszewski J. (2013). Low levels of copper disrupt brain amyloid-β homeostasis by altering its production and clearance. Proc. Natl. Acad. Sci. USA.

[30] Morris M.C., Evans D.A., Tangney C.C., Bienias J.L., Schneider J.A., Wilson R.S., Scherr P.A. (2006). Dietary copper and high saturated and trans fat intakes associated with cognitive decline. Arch. Neurol..

[31] Hill G.M., Brewer G.J., Juni J.E., Prasad A.S., Dick R.D. (1986). Treatment of Wilson’s disease with zinc. II. Validation of oral 64copper with copper balance. Am. J. Med. Sci..

[32] Ohrvik H., Thiele D.J. (2014). How copper traverses cellular membranes through the mammalian copper transporter 1, Ctr1. Ann. N. Y. Acad. Sci..

[33] Brewer G.J. (2012). Copper excess, zinc deficiency, and cognition loss in Alzheimer’s disease. Biofactors.

[34] Ueda K., Kawano H., Hasuo Y., Fujishima M. (1992). Prevalence and etiology of dementia in a Japanese community. Stroke.

[35] White L., Petrovitch H., Ross G.W., Masaki K.H., Abbott R.D., Teng E.L., Rodriguez B.L., Blanchette P.L., Havlik R.J., Wergowske G. (1996). Prevalence of dementia in older Japanese-American men in Hawaii: The Honolulu-Asia Aging Study. JAMA.

[36] Hardy J., Selkoe D.J. (2002). The amyloid hypothesis of Alzheimer’s disease: Progress and problems on the road to therapeutics. Science.

[37] Chartier-Harlin M.C., Crawford F., Houlden H., Warren A., Hughes D., Fidani L., Goate A., Rossor M., Roques P., Hardy J. (1991). Early-onset Alzheimer’s disease caused by mutations at codon 717 of the β-amyloid precursor protein gene. Nature.

[38] Sarell C.J., Wilkinson S.R., Viles J.H. (2010). Substoichiometric levels of Cu2+ ions accelerate the kinetics of fiber formation and promote cell toxicity of amyloid-β from Alzheimer disease. J. Biol. Chem..

[39] Sayre L.M., Perry G., Harris P.L., Liu Y., Schubert K.A., Smith M.A. (2000). *In situ* oxidative catalysis by neurofibrillary tangles and senile plaques in Alzheimer’s disease: A central role for bound transition metals. J. Neurochem..

[40] Nunomura A., Tamaoki T., Motohashi N., Nakamura M., McKeel D.W., Tabaton M., Lee H.G., Smith M.A., Perry G., Zhu X. (2012). The earliest stage of cognitive impairment in transition from normal aging to Alzheimer disease is marked by prominent RNA oxidation in vulnerable neurons. J. Neuropathol. Exp. Neurol..

[41] Lee H.G., Castellani R.J., Zhu X., Perry G., Smith M.A. (2005). Amyloid-β in Alzheimer’s disease: The horse or the cart? Pathogenic or protective?. Int. J. Exp. Pathol..

[42] Brewer G.J., Yuzbasiyan-Gurkan V., Dick R., Wang Y., Johnson V. (1993). Does a vegetarian diet control Wilson’s disease?. J. Am. Coll. Nutr..

[43] Sinha R., Cross A.J., Graubard B.I., Leitzmann M.F., Schatzkin A. (2009). Meat intake and mortality: A prospective study of over half a million people. Arch. Intern. Med..

[44] Mursu J., Robien K., Harnack L.J., Park K., Jacobs D.R. (2011). Dietary supplements and mortality rate in older women: The Iowa Women’s Health Study. Arch. Intern. Med..

