# Correction: Brewer, G.J. Copper-2 Ingestion, Plus Increased Meat Eating Leading to Increased Copper Absorption, Are Major Factors Behind the Current Epidemic of Alzheimer’s Disease. *Nutrients* 2015, *7*(12), 10053–10064

**DOI:** 10.3390/nu8040194

**Published:** 2016-04-01

**Authors:** George J. Brewer

**Affiliations:** Human Genetics, University of Michigan, 3820 Gensley Road, Ann Arbor, MI 48103, USA; brewergj@umich.edu; Tel.: +1-734-761-7970

On page 10055, line 10, of the original publication [1] it was incorrectly stated that “However, this argument is invalid because in 1911, half the population of France was 60 or older”. Instead, this statement should read “However, this argument is invalid because in 1911, half the population of France lived to age 60 or older”.

This change has no material impact on the conclusions of my paper. I apologize to the readers.
